# Conscientiousness increases efficiency of multicomponent behavior

**DOI:** 10.1038/srep15731

**Published:** 2015-10-27

**Authors:** Ann-Kathrin Stock, Christian Beste

**Affiliations:** 1 Cognitive Neurophysiology, Department of Child and Adolescent Psychiatry, Faculty of Medicine of the TU Dresden.

## Abstract

Many everyday situations require the flexible interruption and changing of different actions to achieve a goal. Several strategies can be applied to do so, but those requiring high levels of cognitive control seem to confer an efficiency (speed) advantage in situations requiring multi-component behavior. However, it is elusive in how far personality traits affect performance in such situations. Given that top-down control is an important aspect of personality and furthermore correlates with conscientiousness, N = 163 participants completed the NEO-FFI and performed an experimental (stop-change) paradigm assessing multicomponent behavior. Applying mathematical constraints to the behavioral data, we estimated the processing strategy of each individual. The results show that multicomponent behavior is selectively affected by conscientiousness which explained approximately 19% of the measured inter-individual behavioral variance. Conscientiousness should hence be seen as a major personality dimension modulating multicomponent behavior. Highly conscientious people showed a more effective, step-by-step processing strategy of different actions necessary to achieve a goal. In situations with simultaneous requirements, this strategy equipped them with an efficiency (speed) advantage towards individuals with lower conscientiousness. In sum, the results show that strategies and the efficiency with which people cope with situations requiring multicomponent behavior are strongly influenced by their personality.

Many everyday situations require us to interrupt and chain different actions to achieve a superordinate task goal. When driving a car, for example, you have to coordinate steering, braking, switching gears and tuning the radio. Results from cognitive psychology suggest that in such multicomponent situations, different task goals need to be activated to cascade different actions during multicomponent behavior^1^,^2^,^3^,^4^. In experimental paradigms examining these processes (i.e. stop-change paradigms) it has been shown that people may apply different strategies to cope with situations requiring action cascading and multicomponent behavior^2^: When the requirements for different responses are put up simultaneously, one is left with the decision of whether to processes two actions at once (overlapping/parallel strategy), or whether one prefers processing the actions in a step-by-step fashion (serial strategy), i.e. finishing the first action before turning to the second action. It has been shown that the “overlapping strategy” is related to an inefficient unfolding of behavior as measured by prolonged response times of the involved actions. The main reason for this is that several processes have to share a limited response selection capacity^1^,^2^. By contrast, this is not the case when people process several stimuli in a step-by-step fashion. Using mathematical constraints, it is possible to estimate which strategies subjects apply during action cascading^2^. The stop-change paradigm used in this study allows for the calculation of a slope measure using the so-called SCD-RT slope function^1^,^2^. On a continuum from more serial (i.e. step by step) to more parallel (i.e. overlapping), flatter slope values (closer to 0) reflect a more serial processing mode while steeper slope values (closer to −1) reflect a more parallel (i.e. less efficient) processing mode (for details on the experimental paradigm as well as the calculation and interpretation of the slope parameter, please refer to the methods section). While these findings have been replicated several times^5^,^6^, it became apparent that the inter-individual variance in the strategy applied to cope with situations requiring multicomponent behavior in daily life is high. However, the factors that contribute to these inter-individual differences in the efficiency of multicomponent behavior are elusive and it has never been tested whether personality traits affect the efficiency of multicomponent behavior.

In the current study, we therefore examine in how far personality traits are an important modulator of the strategies applied during multicomponent behavior that plays a major role in daily life. Previous results suggest that cognitive control mechanisms are especially challenged when people are simultaneously confronted with several stimuli or action requirements triggering multicomponent behavior^1^,^7^. Postponing the processing of a task, even though it was presented at the same time as another one, requires top-down behavioral control of the person’s actions. This top-down control of person’s actions is considered to be an important aspect of personality^8^,^9^. The probably most well-known approach to assessing personality are the Big Five (extraversion, openness, conscientiousness, agreeableness, and neuroticism), which have been widely replicated and shown to affects many different aspects of our lives^10^,^11^,^12^. Among other aspects, differences in several personality traits including extraversion and neuroticism seem to affect our cognitive abilities, response speed in various tasks, working memory capacities, task switching, and executive efficiency in both healthy and diseased individuals^13^,^14^,^15^,^16^,^17^,^18^. With regard to our study question, consciousness stands out among the Big Five. It has been associated with measurable skills and aptitudes including self-control, planning or goal-directed and efficient behavior^11^,^19^,^20^,^21^,^22^. Typical items of the NEO-FFI examining conscientiousness are: “I work hard to accomplish my goals” or “I am a productive person who always gets the job done”. Because this top-down control of a person’s actions is considered to be an important aspect of personality^8^,^9^,^23^ and due to the beneficial effects of high conscientiousness^21^, this trait is likely to play an important role in the cognitive control processes driving multicomponent behavior. Even more importantly, executive functions have been suggested to be the primary mechanism by which conscientiousness affects behavior^20^,^24^. Matching this, conscientiousness seems to be the only Big Five factor that correlates with volume differences in the dorsolateral prefrontal cortex, a structure that plays a key role in executive functioning^23^. This is of particular importance in the context of the current study, because action cascading processes important for multicomponent behavior reflect instances of executive control functions. For these processes, it is particularly important to be focused on higher-order goals; i.e. it is important to process the whole chain of actions as efficiently as possible. We therefore hypothesize that conscientiousness is a major factor affecting efficiency of action cascading and multicomponent behavior. Increasing levels of conscientiousness should be related to more efficient unfolding of multicomponent behavior, as reflected by a flatter slope parameter.

## Results

**Figure 1:**
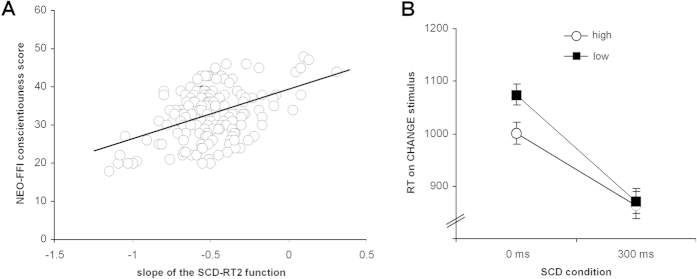
(**A**) Scatterplot showing the correlation between the slope of the SCD-RT function giving an estimate about the strategy applied by each individual during the stop-change paradigm. (**B**) Interaction between a high and low level of conscientiousness and reaction times (RTs) in the different stop-change conditions (SCD0 and SCD300) of the experimental paradigm.

As outlined in the introduction and methods section, the slope of the SCD-RT function provides an estimate of the strategy used during action cascading. The mean slope of the SCD-RT function was −0.49 ± 0.23. To examine the effects of each NEO-FFI dimension (i.e., agreeableness, openness, extraversion, conscientiousness, neuroticism) on the slope of the SCD-RT function (dependent variable), a regression analysis using the “enter method” (i.e., the effect of each of the dimensions was tested in the same model) was calculated. The regression model yielded significant results (F(5,157) = 8.51; p < 0.001) and revealed an R^2^ of 0.213. Within the model, only the factor “conscientiousness” was significant (β = 0.435; t = 5.81; p < 0.001). All other predictors were not significant (all β < 0.112; t < 1.41; p > 0.2). The correlation between conscientious and the slope of the SCD-RT2 function is shown in Fig. 1A. Using only “conscientiousness” as a predictor again revealed significant results (F(1,62) = 39.45; p < 0.001) showing that “conscientiousness” was also a significant predictor (β = 0.444; t = 6.28; p < 0.001) when no other personality dimensions were added in the regression analysis. Notably, the regression coefficient obtained for conscientiousness was the same as in the full model, suggesting that the other personality variables entered into the model did not mediate the effect of conscientiousness.

As can be seen, a flatter slope of the SCD-RT function was related to higher levels of conscientiousness. According to the functional meaning of the slope of the SCD-RT function, this indicates that a higher level of conscientiousness is related to a more serial (i.e. step-by-step) processing strategy during action cascading. In this context, it is important to note that the slope of SCD-RT function as an indicator of the processing strategy has been shown to be most strongly influenced by performance in the SCD0 condition^1^,^2^. As explained in the methods section, this is plausible because only this condition allows subjects to “chose” their individual action cascading strategy. Hence, processing strategy differences related to conscientiousness should primarily modulate CHANGE RTs in the SCD0 condition. To test for this, we calculated a median split on the conscientiousness score. The resulting groups were used as a between-subject factor in a mixed effects ANOVA using SCD0 and SCD300 RTs as within-subject factors. This model revealed a main effect of “SCD interval” (F(1,161) = 542.98; p < 0.001; η^2^ = 0.771) showing that RTs were longer in the SCD0 (1037 ± 15) than in the SCD300 (872 ± 17) condition. More importantly, it also revealed an interaction of “SCD interval x group” (F(1,161) = 16.16; p < 0.001; η^2^ = 0.091) which is shown in Fig. 1B. Bonferroni-corrected post-hoc tests revealed that in the SCD0 condition, subjects with high conscientiousness scores showed shorter RTs than subjects with low conscientiousness scores (t_161_ = −2.41; p = 0.008) (refer Fig. 1B). As expected, there was no such difference in the SCD300 condition (t_161_ = −0.42; p > 0.4). However, it is widely accepted that the use of median split might be problematic because it cuts relevant information, which is not the case for continuous variables. We therefore also added conscientiousness as a covariate to the ANOVA, which revealed an effect of “SCD interval x conscientiousness” (F(1,161) = 39.72; p < 0.001; η^2^ = 0.356). Correlations analyses showed that there was an effect of conscientiousness in the SCD0 condition (r = −352; p = 0.001), but not in the SCD300 condition (r = 0.094; p > 0.1). In sum, this shows that conscientiousness especially affects unfolding of multicomponent behavior and action cascading when subjects are free in their choice how to process different components of a task.

Other than that, there were generally no effects of conscientiousness on the accuracy of responding in the regression analyses using the NEO-FFI dimension or ANOVAs (all F < 1.13; p > 0.3). This demonstrates that the effects obtained for the RT data are unbiased with respect to a speed-accuracy trade-off. Similarly, there was no effect of each of the NEO-FFI dimensions on SSD, SSRT, and GO RTs (all F < 0.54; p > 0.745). These findings did not change when SSD, SSRT and GO RTs were directly correlated with conscientiousness (as a continuous variable) (all r < 0.89; p > 0.2). Adding sex to the ANOVA did not reveal any modulation by this factor (all F < 0.8; p > 0.2), suggesting that sex is not an important variable to consider in the context of this investigated cognitive control function.

## Discussion

In the current study, we examined in how far the Big Five personality dimensions affect performance and efficiency of behavioral control in situations requiring multicomponent behavior. To this end, we used an experimental paradigm that allows to estimate the strategy applied during action cascading and multicomponent behavior on the basis of mathematical constraints^2^. We hypothesized that the trait of conscientiousness should be most important for the modulation of multicomponent behavior since executive functions have been suggested to be the primary mechanism by which conscientiousness affect behavior^20^,^24^.

Matching this, our data shows that conscientiousness affects the processing strategy subjects apply when required to chain different actions in order to achieve a task goal. Inter-individual differences in the processing strategy applied during action chaining were quantified with the help of the SCT-RT function put forward by Verbruggen *et al*.^2^. A flatter slope, which indicates that task goals are activated in step-by-step rather than an overlapping/parallel fashion, was related to higher levels of conscientiousness. A higher level of conscientiousness is hence related to a more pronounced step-by-step processing strategy. The finding that conscientiousness explained approximately 19% of variance of the slope of the SCD-RT function hence suggests that conscientiousness plays a major role in multicomponent behavior and underlines suggestions that conscientiousness is a very important factor in the Big Five personality traits^22^,^25^.

Importantly, differences in the SCD-RT slope measure are mainly determined by differences in SCD0 RTs. Only in the SCD0 condition, participants have the “choice” of whether they want to try to process STOP and CHANGE-related stimuli at the same time, or instead prioritize stopping over changing in a more serial approach^1^,^2^. In order to perform action cascading in an efficient way, it is however essential to prioritize the processing of one stimulus (i.e. the STOP stimulus) over the other (i.e., the CHANGE stimulus). If there is no such priorization of processing and both task goals are activated in parallel, they will most likely overstrain limited response selection capacities, which will consequentially slow down response selection processes. In line with this, it was notably the SCD0 condition that was most affected by differences in conscientiousness: Participants scoring high in conscientiousness had faster RTs in the SCD0 condition than participants scoring low in conscientiousness. By contrast, there were no such significant group differences in the SCD300 condition. This is however logical since the 300 ms delay between STOP and CHANGE stimuli enforces serial processing in all participants, irrespective of their “preferred” action cascading strategy^2^. The results therefore show that high level of conscientiousness are related to a more efficient unfolding of multicomponent behavior when subjects are free in their choice of how to process different components of a task.

Efficient action cascading has been shown to require top-down behavioral control of one’s actions^1^,^6^. For this, it is necessary to behave self-controlled, organized and goal-oriented. These aspects are known to be a major facet of conscientiousness^9^,^20^,^21^,^23^ so that it seems plausible that high levels of conscientiousness are related to a more efficient unfolding of multicomponent behavior. On the other side, individuals lacking self-control and goal-orientation (i.e., those having a low level of conscientiousness) should be more prone to a more inefficient, parallel processing of stimuli and task goals because they are less able to exert top-down behavioral control. Because those individuals are unable to prioritize the activation of one task goal of the other, they are likely to overstrain limited response selection capacities which results in an inefficient unfolding of multicomponent behavior. Furthermore supporting our line of arguments, is has been suggested that conscientiousness contributes to the meta-trait of stability^26^, which has recently been shown to be beneficial in the stop-change task since it promotes a more serial processing of task goals^6^. In this regard, our results fit well into the broader literature on conscientiousness.

It may be seen at odds with the above argumentation that performance in stopping an ongoing response (reflected by the SSRT) was not affected by the level of conscientiousness, even though inhibition is an important aspect of executive control. However, the paradigm does not assess pure inhibition as stopping was always coupled with a changing requirement. In other words, the stop process is only one component of a cascade of actions necessary to achieve a multicomponent task goal. Given that both responses (stopping and changing) depend on a limited response selection capacity and that there will most likely always be some overlap between the involved task goals (even in those applying a more serial strategy, see^1^,^2^,^27^), the stop-change paradigm does not allow for the assessment of “pure” inhibition.

Aside from conscientiousness, none of the other Big Five dimensions (agreeableness, extraversion, neuroticism, and openness) modulated multicomponent behavior. This matches the fact that until now, they have rarely been shown to affect multicomponent behavior via the modulation of executive functions: “Neuroticism” reflects inter-individual differences in how easily we experience unpleasant emotions and is related to aspects of emotional stability. Even though it has been suggested to focus attention^28^,^29^ and/or to induce “cognitive noise” and thereby slow down responding in a number of cognitive tasks^14^, we do not see a reason to assume why or how it would exert a differential effect on the different conditions assessed in this task. The main reason for this is that investigated inter-individual differences in action cascading do not seen to be driven by differences in attentional processing^1^. Furthermore, the effects of neuroticism seem to be rather confined to those aspects of behavior that are influenced by (negative) emotions^23^. Given that we have investigated strictly “cool” cognitive control (as opposed to the functionally and neuroanatomically distinct “hot” cognitive functions involving emotions^30^) our observations do not concern those aspects of executive control. “Agreeableness” reflects interpersonal aspects of being compassionate or cooperative rather than suspicious towards others. Like neuroticism, this trait does not relate to the requirements in the investigated cognitive faculties. “Extraversion” reflects aspects of sociability, assertiveness, and the tendency to seek stimulation. It has repeatedly been associated with differences in dopaminergic neurotransmission^31^, which also plays an important role for the investigated aspects of action cascading^6^. However, those findings seem to be confined to motivational aspects of behavior^31^, which is also underlined by the finding that extraversion correlates with the volume of the medial orbitofrontal cortex, which plays a key role in reward processing^23^. Yet, extraversion has not been associated with “cool” aspects of cognition which are not modulated by reward. Hence, these findings cannot be generalized to the investigated aspects of action cascading. Lastly, “Openness” reflects the tendency to seek stimulation and intellectual curiosity. Like extraversion, it has been associated with changes in dopaminergic neurotransmission^26^ but unlike conscientiousness, it has not been shown to correlate with changes in dorsolateral prefrontal areas^23^. Taken together, “Extraversion” and “Openness” have been shown to positively correlate with cognitive abilities including non-verbal decoding in multitasking situations^13^,^15^ and have been suggested to play a role in error monitoring^32^. Yet, they have not been shown to correlate with the cognitive control functions required for an efficient (i.e. serial) cascading of actions in multicomponent behavior. Hence, these dimensions of the Big Five have little in common with processes relevant to exert a high level of cognitive control.

Yet, differences in Big Five personality traits and the meta-trait of stability have been associated with the dopaminergic and serotonergic systems^26^,^33^,^34^. The question of how differences in personality traits and those neurobiochemical systems interact in modulating action cascading in multicomponent behavior, should probably be subject to further future research on this topic.

In summary, our study shows that multicomponent behavior, which is tremendously important in daily life, is strongly connected to conscientiousness but not any other of the Big Five personality traits. People scoring high in conscientiousness differ in the strategy that they apply to cope with situations requiring multicomponent behavior. More specifically, they process different actions required to achieve a goal in a more step-by-step manner. This processing strategy provides highly conscientious people with an advantage in situations providing us with several options of how to produce and organize multicomponent behavior. By contrast, individuals scoring low in conscientiousness applied a less efficient and more parallel mode of action cascading. These results stress the importance of taking personality traits into consideration when trying to understand factors that modulate cognitive control processes.

## Methods

### Participants

In total, n = 163 subjects between 20 and 25 years of age (mean 24 ± 3.18) participated in the study. There were 99 females and 64 males. The study was approved by the Ethics committee of the TU Dresden and conducted in accordance with the Declaration of Helsinki and each participant gave written informed consent before the start of their study participation. All participants had normal or corrected-to-normal vision and normal hearing abilities. No participant reported any history of neurological or psychiatric disorders. Each participant completed the German version of the NEO-FFI^35^ before completing the experimental paradigm used to assess multicomponent behavior. This employed German version has a high consistency as indicated by Cronbach’s alpha values between α = 0.72 und α = 0.87 for the different scales. Overall, the cohort had a mean score of 31.8 (6.7) in the “agreeableness dimension”, 32.7 (6.7) in the “conscientiousness dimension”, 31.4 (6.2) in the “openness dimension”, 30.4 (6.5) in the “extraversion dimension” 30.4 (6.5), and 19.1 (7.7) in the “neuroticism dimension”.

### Task to examine multicomponent behavior (action cascading)

**Figure 2:**
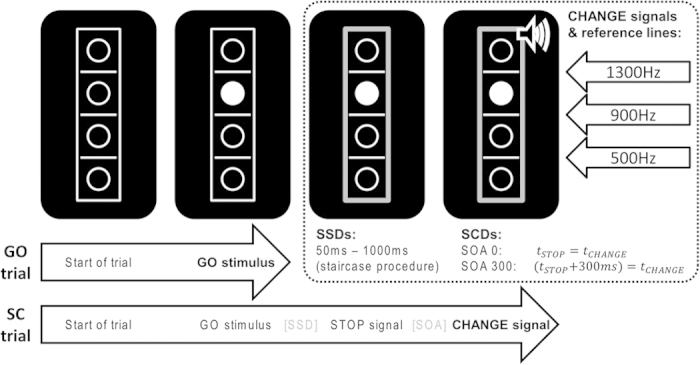
Schematic illustration of the stop–change paradigm as described in the methods section. While a right hand response to the GO stimulus ended GO trials, left hand responses to the CHANGE stimulus ended SC trials. The stop-signal delay (SSD) between the GO stimulus and the STOP signal (red thick frame, depicted grey in this figure) was adjusted by means of a staircase procedure. The stop-change delay (SCD) between the onset of the STOP and CHANGE stimuli was fixed and set to 0 ms in half of SC trials (SCD0 condition) and to 300 ms in the other half (SCD300 condition). The three CHANGE stimuli were associated with one of the three reference lines (see upper right corner). The volume symbol in this graph was drawn by the authors using Microsoft Powerpoint © software.

In order to examine whether aspects of multicomponent behavior may be related to conscientiousness, each participant completed the stop-change task. The task was a modified version of the stop-change paradigm by Verbruggen *et al*.^2^ and is illustrated in Fig. 2.

In a dimly lit and sound-attenuated room, stimuli were presented on a 17′′ CRT monitor and via headphones. Presentation software (v. 14.9, Neurobehavioral Systems, Inc.) was used to present stimuli and record responses. Responses had to be given with four buttons on two custom-made response devices.

Each participant completed an extensive training block prior to the start of the experiment. Starting each trial, a rectangular frame (20 × 96 mm) containing four vertically aligned circular frames (8 mm diameter) and three horizontal reference lines (line thickness: 1 mm; width: 8 mm) separating the circles were presented on black background in the center of the screen. After 250 ms, one of the circles was filled with white color, thus turning into the GO stimulus (target). In the GO trials (two thirds of trials), participants were instructed to indicate the target’s location relative to the middle reference line.

Responses were given by pressing the inner right key with the right index finger (“below” judgment) or by pressing the outer right key with the right middle finger (“above” judgment) on one of the response devices. The stimuli remained on the screen until a response was given. One third of the trials were stop-change (SC) trials. Like GO trials, they started with the presentation of the empty array followed by the target after 250 ms. After a variable stop signal delay (SSD), a STOP signal (a red rectangle replacing the white frame) was presented. Upon its appearance, participants were required to try to inhibit their regular right hand response to the GO stimulus. Initially, the SSD was set to 450 ms and adapted to the participants’ performance by means of a ‘staircase procedure’ yielding a 50% probability of successfully inhibited GO responses^2^,^36^. The STOP signal was followed by a 100 ms sine tone CHANGE stimulus (75 db SPL) presented via headphones. The stop-change delay (SCD) between the STOP and CHANGE signals was either 0 ms (SCD 0) or 300 ms (SCD 300). The CHANGE signal could be either high (1300 Hz), medium (900 Hz) or low (500 Hz), and coded for the high, middle, and low reference line, respectively. Each of the three tones was in effect equally often. The required CHANGE response had to be performed with the left hand and indicate the target’s position relative to the reference line assigned by the CHANGE stimulus. If the target was located above the newly indicated reference line, an outer left key press with the left middle finger was required. If it was below the newly assigned reference line, a left inner key press with the left index finger was required. After each SC trial, the SSD for the next SC trial was adjusted by the above-mentioned staircase algorithm: In case of a “flawless” SC trial as defined by correct inhibition of GO response and a correct CHANGE response, the SSD was prolonged by 50 ms. In case not both of these criteria were met, the SSD was shortened by 50 ms. Due to this adaptive procedure, subjects respond incorrectly or prematurely in roughly half of the SC trials. Yet, SSDs were confined to the range of 50–1000 ms to keep the experiment within reasonable limits. Each trial was followed by a fixed inter-trial interval of 900 ms. Participants were instructed to respond as fast and accurately as possible. In case of response times (RTs) exceeded 1000 ms in GO trials or 2000 ms in SC trials, the German word “Schneller!” (engl. “Faster!”) appeared above the box until the participant responded and thereby ended the trial. The experiment consisted of 864 pseudorandomized trials (i.e. 576 GO trials, 144 SCD0 trials and 144 SCD300 trials) divided into 6 blocks.

### Estimating the strategy of action cascading

Verbruggen *et al*.^2^ were able to demonstrate that three task goals are involved for the required process of action cascading in the stop-change paradigm: responding to the GO signal, stopping the initially intended GO response upon the presentation of the stop signal, and responding to the CHANGE signal.

Using a horse race analogy, it is commonly assumed that the first two processes triggered by the GO and STOP stimuli are largely independent^2^,^36^,^37^. Furthermore, stopping is not influenced by the demands of the GO response it ends since the GO task goal becomes obsolete as soon as the STOP stimulus is introduced^2^,^38^. Hence, there should be little to no unwanted (dual task) interference between the GO and STOP task goals.

However, things lie differently for the STOP and CHANGE task goals because both need to be correctly carried out in order to comply with task requirements. Having both of these task goals active at the same time should be disadvantageous and result in an order-control problem because the STOP process needs to selectively terminate the GO process, but not the CHANGE process^2^,^39^. By contrast, a sequential activation of these task goals would not necessitate this kind of selective and cognitively challenging inhibition, consequentially increasing processing efficiency.

Based on these assumptions, the strategy used to cascade these two different task goals and actions was estimated using a mathematical model. As described above, the paradigm introduces two different SCD intervals. The SCD0 condition provides the participants with a “choice” of how to process the STOP- and CHANGE-associated processes. Bottleneck models of response selection capacity limitations suggest that response selection can be performed either serially (i.e., one step/task goal is executed after another) or in parallel (i.e., steps are processed in parallel so that there is a temporal overlap of processes/task goals)^2^,^27^. Assuming that response selection depends on a restricted resource and that a parallel activation of task goals requires a more complex and selective inhibition strategy, the strategy used to cascade STOP and CHANGE processes should result in longer CHANGE response RTs in parallel than in serial processing . If participants choose to simultaneously process STOP- and CHANGE-associated task goals (i.e., in parallel), RTs increase because these processes must share a limited capacity and the STOP process may furthermore obstruct the CHANGE response. However, the participants can also choose to process STOP- and CHANGE-associated task goals in a step-by-step (i.e., serial) manner. In this case, the STOP process is finished before the CHANGE process so that they will not have to share a limited capacity and cannot interfere with each other. This ultimately results in shorter CHANGE response RTs than the parallel/simultaneous strategy.

Critically, the SCD300 condition enforces serial processing because the STOP process has usually finished when the CHANGE stimulus is presented 300 ms later^2^. Therefore, the SCD300 condition provides a baseline measure for CHANGE response RTs in a purely serial cascading strategy. If a parallel processing strategy has been used, RTs are substantially longer in the SCD0 than in the SCD300 condition. If a serial processing strategy has been used, the RTs are less prolonged and more similar to those in the SCD300 condition. The ratio of RT differences in the two SCD conditions therefore gives an estimate of the strategy used during action cascading in the SCD0 condition^2^:


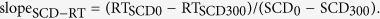


This slope value was individually calculated for each participant. When the STOP process has not finished by the time the CHANGE process is initiated (parallel processing strategy), the slope value becomes larger (i.e., more negative), indicating that action cascading is less efficient. If the STOP process has finished (serial processing strategy), the slope becomes less negative, indicating that action cascading becomes more efficient^2^. Obtaining a mean slope value between 0 and −1 therefore suggests that some (but not all) of the CHANGE response processes were initiated prior to the termination of the inhibitory process stopping the GO response. Therefore, the slope of the SOA-RT function is flatter in the case of more efficient processing than in the case of the less efficient processing mode.

## Additional Information

**How to cite this article**: Stock, A.-K. and Beste, C. Conscientiousness increases efficiency of multicomponent behavior. *Sci. Rep*. **5**, 15731; doi: 10.1038/srep15731 (2015).
